# The Causes for Lack of Interest to Blood Donation in Eligible Individuals, Mashhad, Northeastern Iran

**Published:** 2012-01-01

**Authors:** M T Shakeri, A Vafaee, H Esmaeily, N Shafiei, R Bazargani, ME Khayamy

**Affiliations:** 1 Department of Biostatistics, Mashhad University of Medical Sciences, Mashhad, Iran; 2 Center for Health Sciences Research, Mashhad University of Medical Sciences, Mashhad, Iran; 3 Lecturer English Language, Mashhad University of Medical Sciences, Mashhad, Iran; 4 Blood Transfusion Organization of Khorasan Razavi, Mashhad, Iran

**Keywords:** Blood donation, Lack of interest, Knowledge, Attitude

## Abstract

**Background:**

Donor recruitment and retention are significant problems in blood collection agencies around the world. The Aim of this study was to determine the causes of lack of interest to blood donation in eligible individu­als in Mashhad, Northeast of Iran.

**Methods:**

This was a descriptive study. Cases were 1130 non-donor individuals. Participants were selected from eligible individuals in different regions of Mashhad. In this study, surveys included information about age groups, gender, residence area, marriage, education; living situation and job as background variables.

**Result:**

Less than 30% of the cases had enough knowledge about blood donation. There was a significant rela­tionship between location, age, education, occupation and social status with knowledge of blood donation, but there was not a correlation between gender and marital status.

**Conclusion:**

There are some factors which affect the decision for blood donation. There is a need to change the negative attitude by increasing the knowledge considering the individual and the social status.

## Introduction

Donor recruitment and retention are significant problems in blood collection agencies worldwide.[1] In 2006, 1,670,000 blood units were donated in Iran; 48% of them belonged to frequent donors, 42% were among those who donated for the first time and the others were related to those with a previous history of donation.[2] Despite the decrease in population growth rate and advent of modern methods of treatment in medical sciences, the need for blood and of course the new healthy blood donors, is still increasing.[3] Data from the American National Red Cross indicates that the average volunteer donation was about 1.7 times/year[4] and Only 3.5% of the eligible population within Australia donated blood or blood products1,[2] and approximately 60% of new Australian blood donors returned within 2 years for a further donation.[5] Researches showed that certain factors motivated donors to continue donation.[6]

It was shown that most people were never volunteer to donate blood or they stopped donating for unknown reasons.[7][8] Limited numbers of surveys were done on psychological aspects for lack of interest to donate blood.[9] Recognition of effective factors on this category can act as an efficient agent in the alternation of “new methods to attract donors” and increase their effectiveness.[10] This study tried to identify the diverse effective factors on “lack of interest to donate blood” and plan the future programs.

## Materials and Methods

This was a descriptive study. Participants were selected from eligible individuals in different regions of Mashhad, Iran. Educated interviewers filled up the questionnaires. Cases were 1130 non-donor individuals who possessed the general condition, i.e, 17-65 years of age range, more than 50 kg weight that were mentally and physically healthy. After two sessions of education, the interviewers became familiar with questionnaire filling method and the study goals. Those with no history of blood donation in the last 5 years were recognized as non-donor but accepted to enter the study. Participating and filling the questionnaires were optional. Questionnaires were designed according to the study’s purposes and given to authorities and experts to determine its validity regarding different sources and referring to books, magazines, and publications and reliability was confirmed according to Cronbach method among 20 subjects. Cronbach coefficient for knowledge questionnaire and attitude questionnaire were 75% and 72% respectively. The questionnaire included characteristics (social and educational) and also their knowledge (6 questions), attitudes (6 questions), which were measured according to Likert. The addition scores of knowledge and attitude were considered as the total scores for each person. Fifty percent of the total score was determined as weak, 50%-70% as moderate and more than 70% as good.

The data was gathered from all 11 regions in Mashhad, Iran based on the municipallty regions. Stratified-cluster sampling method was used and the municipality region was considered as stratified ad in each municipal region, clusters were chosen. The evaluated variables included age groups, gender, residence area, marriage, education, living condition and job as background variables. The house places were recorded according to municipality region.

Statistical analysis was performed with SPSS software (Version 15, Chicago, IL, USA). All variables were presented as number and percentage. Associations between factors were analyzed by X(2) test and Spearman correlation. P value < 0.05 was considered statistically significant.

## Results

1130 subjects participated in this study among them 969 (85.8%) cases had never donated blood, 161 (14.2%) of them had donated blood for at least one time during the last 5 years. 779 (68.9%) participants were familiar with blood donation. 327 (28.9%) knew the need about blood donation for patients and 942 (83.4%) showed a desire to donate blood.

The donors had little knowledge (11.7%) about the conditions of blood donation. The participants' knowledge about the place to donation (bases in the center of the city) was somehow acceptable (29.3%).

Our findings showed that there was no significant relationship between their knowledge and age groups, gender, marriage and living condition (p>0.05), ex­cept for the living area (p=0.003), education (p<0.001) and job (p=0.001). Unlike their knowledge, in most cases, their attitude was not positive; such as participant's attitude in relation to side effects of blood donation (6%), attitude toward the stimulating factors (3.5%) and attitude toward the time to donate blood (12.4%). Findings revealed that the attitude about deterrent factors was negative but positive for persuasive factors. Findings showed that most impor­tant factors which caused the idea of not being able to donate blood were related to the lack of information about physical condition (67%), anemia (66.85%), the probability of imbalance blood pressure (59.4%), physical weakness (59.3%), diseases (52.3%), malnu­trition (51.5%) and inaccessibility to blood donation bases (51%).

There was no significant relationship between people's beliefs about blood donation obstacles and background variables (except for the living area); however, the participants' general knowledge was effective.

In general the participants considered the blood donation as a positive manner but the possibility of infections after blood donation was the negative evaluation and this had no correlation to any back­ground variables (p>0.05).

Neither the participants' knowledge nor their ex­perience of donation was the modifier items for nega­tive evaluation (p>0.05). Positive evaluation was re­lated to gender (p=0.01), marriage (p=0.004), educa­tion (p=0.02) and job (p=0.002) but not to the living area and age groups (p>0.05). There was a relation­ship between the level of knowledge and the level of attitude to each other (p=0.002).

## Discussion

Lack of knowledge in most cases, the relation between the knowledge level and blood donation, and familiarity with blood donors help people to gain their information via direct referral to blood transfer organizations or through the small groups of donors demonstrating the inadequate information about blood donation in the society.[11] It seems that the methods of blood transfer organizations should promote the knowledge of people about blood donation.[12] The blood donors are informative references for others and they can attract new donors. Thus, education of programs is recommended for heightening awareness.[3] As it was mentioned, more than 69% of participants thought the blood donation may endanger their health and more than 67% of them did not know much about the blood donation process.

On the other hand, participants were shown not to be aware of their eligibility for blood donation, so we recommend providing the essentialities of people’s self-efficiency. Based on previous finding, the participants’ attitude toward blood donation was positive, except for the deterrents.[13] As all the repliers were non-donors, they had encountered attitude conflictions. This finding can be the same as Cacioppo and Gardner' results.[14] In a previous study, the knowledge about the need for blood donation was an important determinant factor,[15] while most of the participants were aware of the need and they knew that it was not limited to a specific time or patient.

So despite the incorrect belief about the conditions of blood donation, the deficiencies in their complementary information have led to the lack of interest for blood donation. Thus, to attract new donors, we should not use the substitute blood but the need for healthy blood donation is important. Also, it is essential to increase the knowledge concerning the time and place which can be regarded as barriers. Like some other studies, our findings revealed that family and residence area indirectly affected other items. We had the problem of insufficient knowledge about blood donation, which decreased the effect of society on individuals. Therefore, the more knowledge in the society, the more improvement of blood donation would be noticed. This will enhance the social and personal tendency. To summarize, it should be clarified that blood donation is a complicated decision influenced by many factors.[13] These factors are different in various societies influencing the tendency for blood donation (directly or indirectly).[16] Attracting new donors depends on the recognition of influential factors, eliminating obstacles and the balance in providing positive behavioral patterns. Experts, in their plans and acts, have to pay attention to all effective factors and identify the problems and demands. These studies result in identifying effective factors. Furthermore, they can become a basic factor in attracting new donors by creating a relationship between different groups of society as well as communication between the participants.
